# Increased expression of fatty acid binding protein 4 and leptin in resident macrophages characterises atherosclerotic plaque rupture

**DOI:** 10.1016/j.atherosclerosis.2012.09.037

**Published:** 2013-01

**Authors:** K. Lee, M. Santibanez-Koref, T. Polvikoski, D. Birchall, A.D. Mendelow, B. Keavney

**Affiliations:** a Institute of Genetic Medicine, International Centre for Life, Newcastle University, NE1 3BZ Newcastle-upon-Tyne, UK; b Institute for Aging and Health, Newcastle University, Newcastle-upon-Tyne, UK; c Regional Neurosurgical Centre, Royal Victoria Infirmary, Newcastle-upon-Tyne, UK

**Keywords:** Plaque rupture, Gene expression, Macrophages, Microarray, Laser micro-dissection

## Abstract

**Objective:**

Resident macrophages play an important role in atheromatous plaque rupture. The macrophage gene expression signature associated with plaque rupture is incompletely defined due to the complex cellular heterogeneity in the plaque. We aimed to characterise differential gene expression in resident plaque macrophages from ruptured and stable human atheromatous lesions.

**Methods and results:**

We performed genome-wide expression analyses of isolated macrophage-rich regions of stable and ruptured human atherosclerotic plaques. Plaques present in carotid endarterectomy specimens were designated as stable or ruptured using clinical, radiological and histopathological criteria. Macrophage-rich regions were excised from 5 ruptured and 6 stable plaques by laser micro-dissection. Transcriptional profiling was performed using Affymetrix microarrays. The profiles were characteristic of activated macrophages. At a false discovery rate of 10%, 914 genes were differentially expressed between stable and ruptured plaques. The findings were confirmed in fourteen further stable and ruptured samples for a subset of eleven genes with the highest expression differences (*p* < 0.05). Pathway analysis revealed that components of the PPAR/Adipocytokine signaling pathway were the most significantly upregulated in ruptured compared to stable plaques (*p* = 5.4 × 10^−7^). Two key components of the pathway, fatty-acid binding-protein 4 (FABP4) and leptin, showed nine-fold (*p* = 0.0086) and five-fold (*p* = 0.0012) greater expression respectively in macrophages from ruptured plaques.

**Conclusions:**

We found differences in gene expression signatures between macrophages isolated from stable and ruptured human atheromatous plaques. Our findings indicate the involvement of FABP4 and leptin in the progression of atherosclerosis and plaque rupture, and suggest that down-regulation of PPAR/adipocytokine signaling within plaques may have therapeutic potential.

## Highlights

► We examined gene expression in macrophages from stable and ruptured plaques. ► The PPAR/adipocytokine signaling pathway was upregulated in ruptured plaques. ► FABP4 and Leptin were highly expressed in ruptured atheromatous plaque macrophages. ► Down-regulation of PPAR/adipocytokine signaling may have therapeutic potential.

## 1 Introduction

Atheromatous plaque erosion and rupture leading to atherothrombotic occlusion or distal embolisation is responsible for the majority of the acute morbidity and mortality of atherosclerosis, such as myocardial infarction, unstable angina and thromboembolic stroke [1]. Differences in cellular composition between stable and ruptured plaques are well established. The macrophage is central to the local inflammatory and apoptotic processes leading to plaque instability and rupture, however, the molecular pathways in macrophages that contribute to plaque rupture are incompletely characterized. The presence and character of differences in gene-expression patterns between macrophages in stable and ruptured lesions could identify metabolic and regulatory pathways that influence plaque instability and rupture. Many previous gene expression studies in human samples have compared whole plaques with normal tissue, while fewer have compared gene expression between stable and ruptured plaques [2–10]. The use of whole plaques for gene expression analysis effectively pools the RNA of various cell types in the plaque relative to their abundance, adding a potentially confounding variable to the analysis. A cell-specific approach has the potential to address the question of gene expression differences between particular cell types in stable and unstable plaques with greater precision than approaches based on the study of whole plaques. Using laser micro-dissection, we isolated total RNA from macrophage-rich regions of stable and ruptured human atheromatous plaques derived from carotid endarterectomy samples which were comprehensively characterized using clinical, radiological and histological criteria, and carried out genome-wide gene expression profiling using microarrays.

## 2 Materials and methods

### 2.1 Specimens

Carotid endarterectomy specimens were obtained from patients undergoing surgery for symptomatic or asymptomatic carotid stenoses at the Regional Neurosurgical Centre, Newcastle-upon-Tyne. Magnetic resonance imaging (MRI) of the brain and 3D gadolinium-DTPA contrast-enhanced magnetic resonance angiography (MRA) of the carotid arteries were performed on a 1.5 T scanner (Intera, Philips Medical Systems). Specimens were snap-frozen in liquid nitrogen in the operating theatre immediately upon removal. A portion of each specimen was sent for histopathological analysis, and classified by two independent observers (KL and TP) according to the Virmani scheme [1]. Informed consent was obtained from all patients and Local Research Ethics Committee approval was granted for this study.

We selected contrasting ruptured and stable samples for RNA analysis. The criteria for ruptured samples comprised all three of the following: symptoms consistent with stroke or transient ischaemic event (TIA) within the last 3 months; significant irregularities of plaque surface on 3D MRA (defined as depressions in the plaque surface of at least 2 mm); and histology of a Ruptured Thin Fibrous Cap Atheroma with thrombus present. Conversely, the criteria for stable samples were: no symptoms attributable to CVA/TIA at any time; a smooth plaque surface morphology on 3D MRA and no evidence of cerebral infarction on MRI; and histology of a thick Fibrous Cap Atheroma or Fibro-Calcific Plaque.

### 2.2 Laser micro-dissection (LMD) and microarray analysis

Cryosections of 10 μm thickness were mounted on RNase-free treated Leica thermoplastic membrane slides, then fixed in 75% ethanol, stained with haematoxylin, dehydrated in increasing gradients of ethanol and rendered RNA stable for laser micro-dissection. At every 10 sections, 3 additional ‘scout’ sections were stained with haematoxylin and eosin to identify anatomical features; these sections were immuno-stained using antibodies to smooth muscle actin (Dako, 1A4, 1:400) to identify regions rich in vascular smooth muscle cells, and CD68 (Dako, PG-M1, 1:125) to identify regions rich in macrophages, using the Vectastain Elite ABC Kit. These sections were used to guide laser micro-dissection of macrophage-rich regions performed on the Leica AS LMD instrument. Macrophage-rich CD68+ regions underlying thick fibrous caps and overlying atheromatous cores of stable lesions, and macrophage-rich CD68+ regions underlying thin fibrous caps, overlying atheromatous cores and adjacent to the defect/rupture of unstable lesions were microdissected. Cellularly mixed regions containing both macrophage and SMCs were avoided.

RNA was isolated using the Qiagen RNeasy Micro kit. RNA quality and quantity were assessed by RIN score using the RNA6000 Pico Labchip (Agilent Bioanalyser 2100, Agilent). Only samples with RIN scores of 6 and above were considered suitable for microarray analysis. For microarray analyses, 20–60 ng of total RNA were subjected to two cycles of linear amplification using the Affymetrix GeneChip Two-Cycle Target Labeling Kit and hybridized to Affymetrix U133plus2 chips. Five ruptured and six stable samples underwent microarray analysis. To replicate the results of the microarray analysis for the most significantly differentially expressed genes, an additional seven stable and seven ruptured samples underwent laser microdissection, and RNA extraction and quality assessment, using the same protocol.

### 2.3 Quantitative PCR

The most significantly differentially expressed genes from the microarray experiment were confirmed using qPCR both in the samples from the microarray experiment and the replication set. Unamplified total-RNA was reverse transcribed to cDNA by random hexamer primers using the SuperScript3 First Strand Synthesis System for RTPCR (Invitrogen). Quantitative real-time PCR was performed using Taqman Gene Expression Assay primer-probes with Taqman Universal PCR Master Mix on the ABI 7900HT platform (Applied Biosystems). Expression levels of succinate dehydrogenase complex subunit-A (SDHA) and peptydylprolyl isomerase-A (PPIA) were used as references. These 2 control genes were selected and validated from 7 candidate genes (details in Supplemental materials). The analysis was performed using the relative quantitation method with PCR efficiency corrections on QBase [11].

### 2.4 Immunohistochemistry

Sections of 5 μm thickness from formalin-fixed paraffin-embedded carotid atheromatous plaque were immunostained with anti-leptin mouse monoclonal antibodies (ABCAM, BDI142, 1:500) and anti-fatty-acid binding-protein 4 (FABP4) rabbit polyclonal antibodies (ABCAM, 1:150) using the Vectastain Elite ABC Kit and visualized using Di-Amino Benzidine, to confirm the expression domains of Leptin and FABP4.

### 2.5 Statistical analysis

The microarray expression profiles were analysed using the GeneSpring GX 7.3.1 analysis package. Raw microarray signal data were pre-processed and normalized using GCRMA. Probesets that were called absent in more than 6 of the 11 samples were excluded from subsequent analyses. To confirm that our LMD protocol had successfully isolated RNA from macrophage-rich regions, we compared the microarray data with expression profiles from the NCBI Gene Expression Omnibus (GEO) repository (www.ncbi.nlm.nih.gov/geo/). These included data from a panel of 79 different cell and tissue types [12] (*n* = 158), from adipocytes [13] (*n* = 24) and from 4 separate macrophage experiments [14–17] (*n* = 106). Relationships were visualized by Condition Tree clustering (using Spearman correlation, as the similarity measure and confidence levels were assessed using 100 bootstraps), and Principal Component Analysis (PCA). Additionally, a list of genes differentially expressed during macrophage activation was obtained from the paper by Cho et al. (3815 genes) [14]. The expression profiles of the micro-dissected specimens for this subset of genes were compared with repository samples of blood cell origin as well as activated and unactivated macrophages.

Differentially expressed probesets were identified using ANOVA. We made allowance for multiple testing using the false discovery rate [18], adopting a threshold FDR of 10%. The BiNGO plug-in (Biological Network Gene Ontology tool) for the open-source Java platform Cytoscape [19] was used to identify significantly over-represented Gene Ontology Biological Processes among the differentially expressed genes. We used WebGestalt [20] (WEB-based Gene SeT AnaLysis Toolkit) to identify among the pathways in the Kyoto Encyclopedia of Genes and Genomes (KEGG) database those that were significantly over-represented among the set of differentially expressed genes. The significance of over-representation was calculated using the hypergeometric test.

## 3 Results

### 3.1 LMD yielded cells with macrophage gene expression profile

**Fig. 1:**
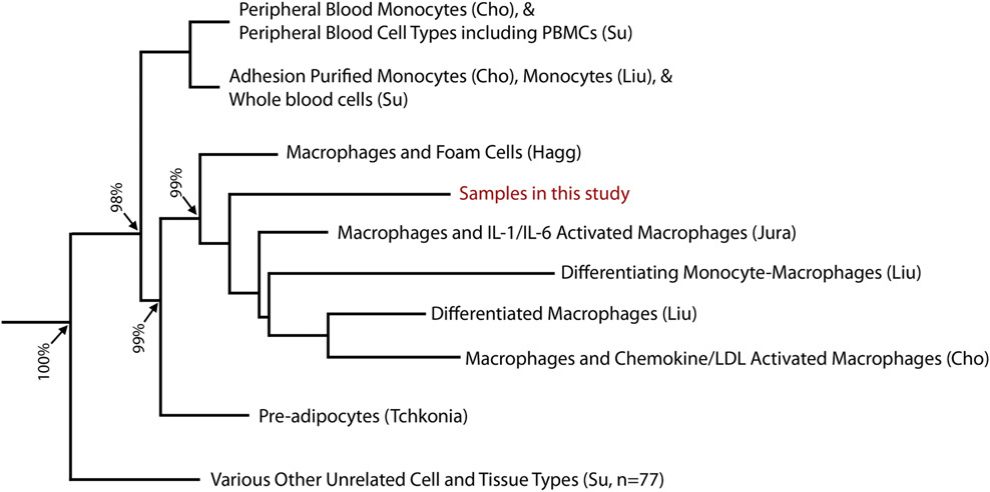
Condition tree dendrogram demonstrating the correlation of the genomic expression profiles of the 11 laser micro-dissected samples to other samples of various types (*n* = 299) using Spearman correlation of the whole array genome-wide gene expression data with bootstrap confidence levels.

The demographics of the 25 patients included in the study (twelve ruptured and thirteen stable samples) are shown in Table 1. Examples of ‘scout’ sections stained with CD68 to identify macrophage-rich regions for LMD and adjacent laser microdissected cryosections are shown in Supplementary Fig. S1. The quality of RNA extracted from the specimens was high; a representative electropherogram of the extracted RNA is shown in the Supplementary Fig. S2. Comparison of the microarray gene expression signatures of the micro-dissected cells to the expression profiles of various cell and tissue types by condition tree analysis showed a close relationship between the micro-dissected cells and macrophage cell-lines with a 99% bootstrap confidence level (Fig. 1). Importantly, the analysis showed only distant relationships to the profiles of other cell types present in the vessel wall, including smooth muscle cells and T-lymphocytes, with a bootstrap confidence level of 100%. The principal components analyses comparing only the expression of genes involved in macrophage activation also showed that the gene expression profiles of the micro-dissected samples were characteristic of activated macrophages (Supplemental Fig. S3).

### 3.2 Differences in gene expression profiles between stable and ruptured plaques

**Fig. 2:**
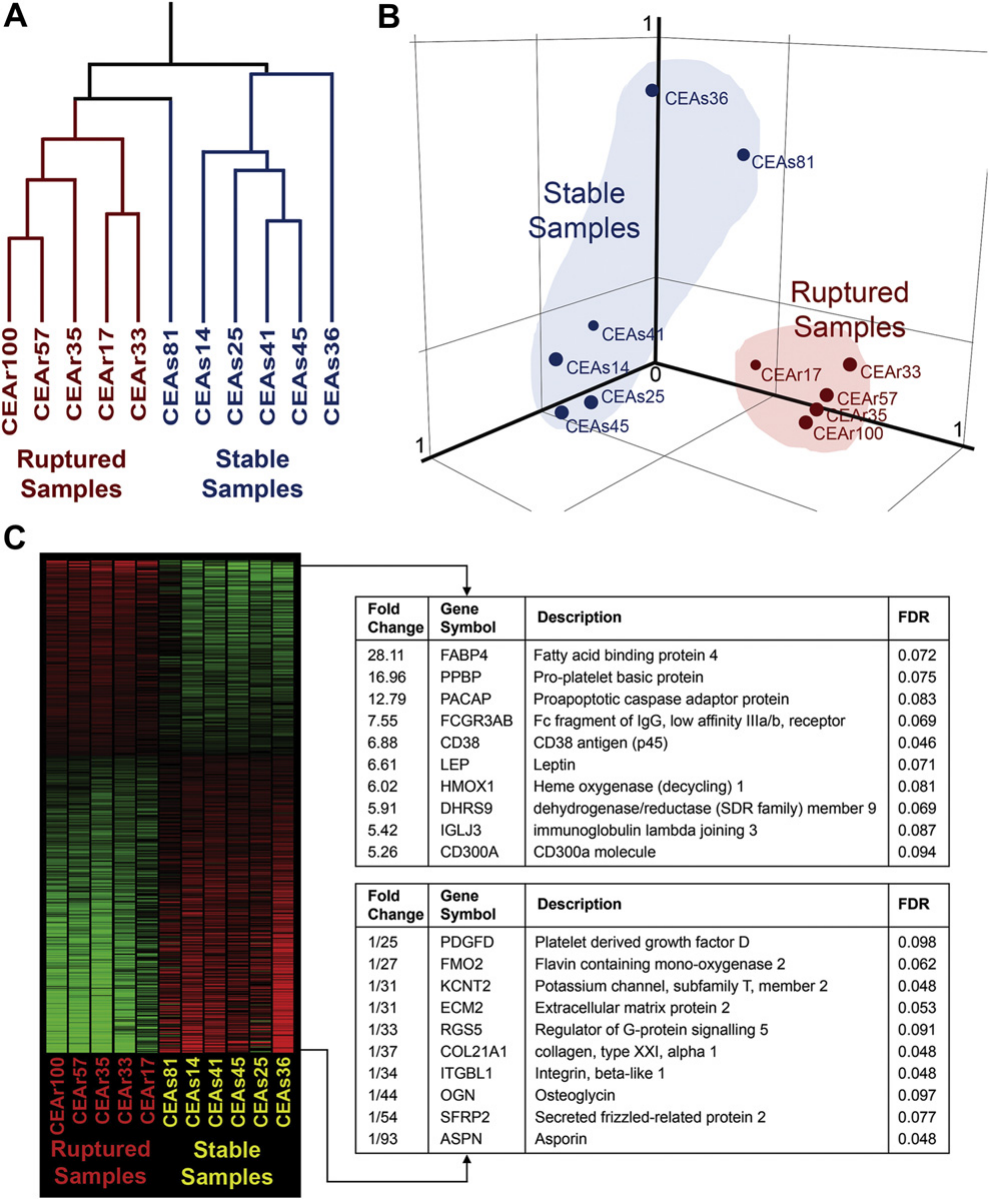
Unsupervised explorative clustering on the entire array datasets of the samples using condition tree (Panel A) and principal components analysis (Panel B). The ruptured group cluster closely together; the stable group, although clearly separate from the ruptured group, has a greater spread (as might be expected in a histologically more heterogeneous group). In panel C, statistically significant differentially expressed genes (1187 probesets representing 914 different genes) ranked by fold difference and represented in a heatmap is shown. Red represents over-expression, green represents under-expression. The top 10 differentially expressed genes from both ends are listed. Fold change values above 1 represents higher relative expression in the ruptured group and values below 1 represents higher relative expression in the stable group. (For interpretation of the references to colour in this figure legend, the reader is referred to the web version of this article.)

**Fig. 3:**
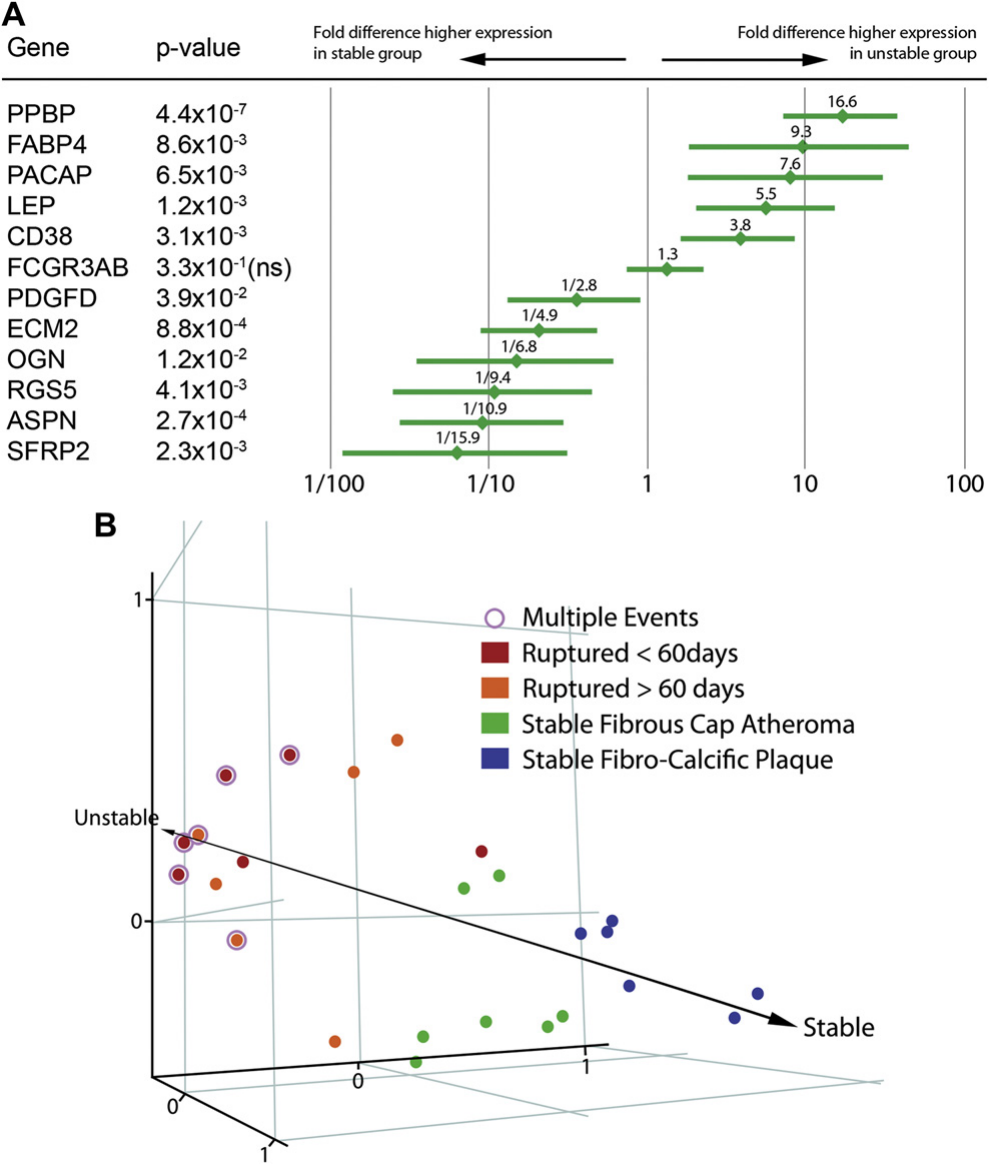
Panel A shows the real-time qPCR results of the 12 most significantly differentially expressed genes tested in 12 ruptured and 13 stable specimens. The fold difference with the 95% confidence intervals and the ANOVA *p*-values are shown. Panel B shows the relative clustering and separation of the 25 samples in gene expression space using principal components analysis on all the genes tested in RTqPCR.

Explorative clustering on the entire dataset using condition tree and principal components analysis showed clustering of the 11 samples into 2 identifiable groups (Fig. 2). These groups corresponded to their clinical designations as stable or ruptured plaques, demonstrating that there were specific differences in the macrophage gene expression profiles between the 2 groups. We identified 914 genes (represented by 1187 probesets) that were significantly differentially expressed in stable and ruptured plaques at an FDR of 0.10. Fig. 2 shows the heatmap together with a list of the twenty genes showing the highest differences in fold change between stable and ruptured samples. A list of the 1187 significantly up- and down-regulated probesets in the microarray analysis is available in the Supplemental materials. The complete MIAME-compliant microarray dataset is available on GEO (http://www.ncbi.nlm.nih.gov/geo/query/acc.cgi?acc=GSE41571). Real-time quantitative PCR assays of the 12 statistically significant genes with the highest fold change, using the unamplified total-RNA from micro-dissection, showed that the correlation between expression levels determined from microarray (linear-amplified) and from real-time PCR (unamplified) was high (Pearson *r* = 0.92), suggesting no significant artefact had been introduced by the two rounds of linear amplification necessary to generate sufficient input material for microarray analyses from the small amounts of RNA obtained from LMD. Expression of these 12 genes was assessed in additional 14 samples, selected and processed in an identical fashion, by real-time PCR. In the combined set of 25 samples (12 ruptured and 13 stable) there was significant evidence of differential expression (at *p* < 0.05) for 11 out of the 12 genes tested (Fig. 3). These results are broadly in accordance with the FDR of 10% that we specified in the analyses of the microarray data. Principal Components Analysis using the real-time PCR data based on the expression of these 12 genes revealed a separation of the 25 samples along an “axis of instability-stability” in gene expression space (Fig. 3) that corresponded to clinical presentation. Samples from patients who had had multiple events clustered at one extreme and samples from patients who were asymptomatic clustered at the other.

### 3.3 Pathway analysis

The most significant Gene Ontology Biological Processes represented in the dataset were cell signaling (with sub-categories- cell communication, 206 genes, *p* = 3.4 × 10^−9^ and signal transduction, 156 genes, *p* = 3.6 × 10^−5^) and cell adhesion (with 61 genes, *p* = 8.1 × 10^−11^). Ten KEGG pathways were significantly differentially expressed at *p* < 0.001 (Supplementary Table S4). Seven of these pathways were chiefly up-regulated in stable samples, two had a balanced configuration, and one, the PPAR/adipocytokine signaling pathway, was chiefly up-regulated in ruptured when compared to stable samples (*p* = 5.4 × 10^−7^). The PPAR/adipocytokine pathway was the fourth most significantly differentially expressed KEGG pathway, after Focal Adhesion (*p* = 1.3 × 10^−10^), Adherens Junction (*p* = 1.4 × 10^−10^), and Actin Cytoskeleton (*p* = 2.6 × 10^−9^) pathways, which were more highly expressed in stable samples. Fifteen differentially expressed genes were present in the PPAR/adipocytokine signaling pathway, of which 11 were up-regulated in the ruptured samples (Supplementary Table S5). FABP4 and Leptin are key genes in the PPAR/adipocytokine signaling pathway; these genes were confirmed by real-time PCR amongst the most differentially expressed statistically significant genes in the ruptured plaques compared to the stable plaques (FABP4: 9.3 fold over-expressed, *p* = 0.009; Leptin: 5.5 fold over-expressed; *p* = 0.001; Fig. 3).

### 3.4 Immunohistochemistry of leptin and FABP4

**Fig. 4:**
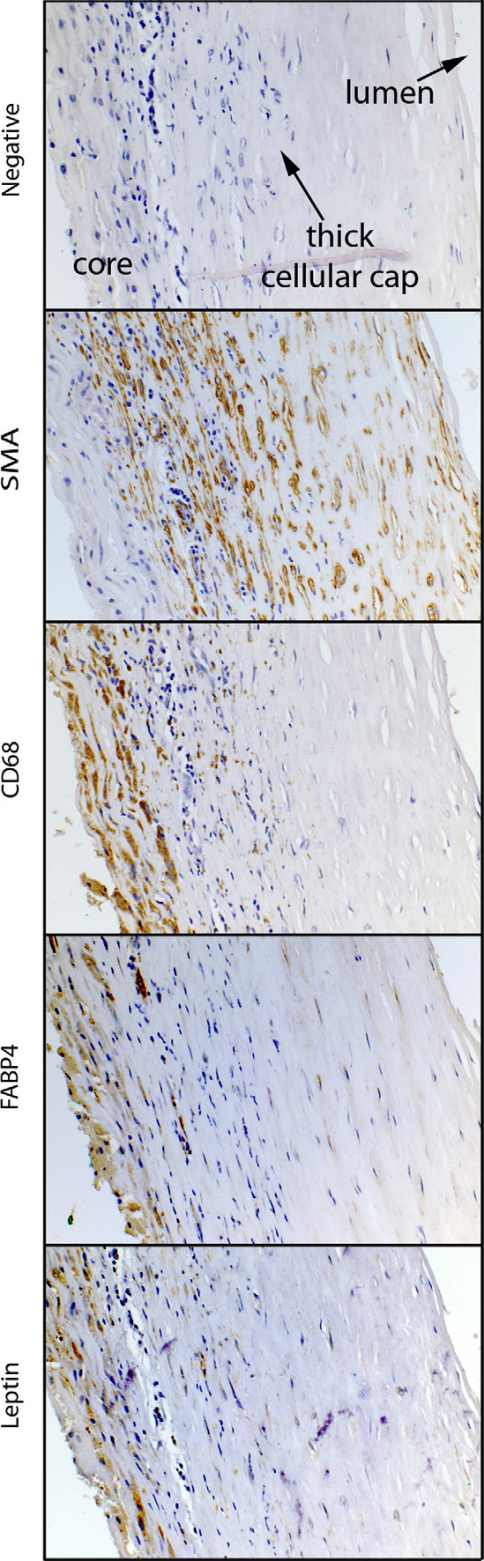
Immunostained paraffin sections of a representative stable atheromatous plaque with a thick fibrous cap. Corresponding negative controls stained with haematoxylin, smooth muscle cells labelled by smooth muscle actin, macrophages labelled by CD68. Leptin and FABP4 immunostaining correlates with the CD68 immunostaining for macrophages.

We carried out immunohistochemical analyses of the proteins encoded by leptin and FABP4 genes to confirm the expression of these genes in plaque macrophages in sixteen of the samples. Leptin and FABP4 staining were present and co-localised to CD68 positive macrophage regions, and were absent from the smooth muscle cell areas (Fig. 4).

## 4 Discussion

This study has demonstrated significant differences in the gene expression profiles between macrophage-rich regions derived from stable and ruptured atheromatous plaques. Comparison with publicly available expression profiles from a variety of cell types showed a close correspondence between our samples and activated macrophages, indicating that we had largely been successful in isolating macrophage-specific RNA. Leptin and FABP4, which both have functions linking lipid metabolism to inflammation, were among the most differentially expressed individual genes. Moreover, the adipocytokine/PPAR signaling pathway incorporating both FABP4 and leptin was the most strongly up-regulated KEGG pathway in ruptured samples (*p* = 5.5 × 10^−7^). This is the first report of the involvement of the adipocytokine/PPAR signaling pathway in plaque rupture. These cell-specific data from clinical samples show the importance of genes in the pathway linking lipid metabolism to inflammation in plaque instability, and confirm the data from mouse models suggesting that modifying the expression or action of these genes in plaque macrophages may have therapeutic potential.

The majority of previous studies quantifying gene expression in human atherosclerosis using microarrays and other approaches have compared atheromatous with normal vessels (reviewed in Bijnens et al. ATVB 2006 [2,3]). While such comparisons are clearly of interest, they are not specific for the stage in the disease process that causes the majority of the acute morbidity and mortality of atherosclerosis, that is, plaque rupture. Fewer studies have been conducted on whole stable and unstable/ruptured plaques [4–10]. While this approach should yield greater specificity for the pathophysiological process of plaque rupture, the results could still be to a degree confounded by the differences in cell composition between stable and ruptured lesions. Indeed, genes that would be correlated with the quantity of macrophages present in a lesion (for example inflammatory mediators, matrix metalloproteinases, pro-apoptotic factors) have been consistently reported as differentially expressed in such studies of whole stable and ruptured plaques [4–10]. For example, Puig et al. identified a gene expression signature of inflamed whole plaques which closely overlapped the expression profile of laser microdissected macrophages from a single plaque [10]. No previous study has compared laser microdissected macrophages from stable and unstable plaques. However, a degree of overlap between our results and those of previous studies of whole ruptured and stable plaques provides external validation for our approach. For example, Papaspyridonos and colleagues' study [6] of ruptured versus stable regions of carotid plaques identified 170 differentially expressed genes, of which 33 were also identified in the present study; and other genes that were highly differentially expressed in our study (for example myeloid-related protein 14 and heme-oxygenase 1) have also been identified by previous reports [7,21]. Dahl and colleagues compared gene expression in whole plaques from symptomatic and asymptomatic stroke patients, without further radiological or histological classification [9]. Among 136 differentially expressed genes, they found FABP4 and leptin to be upregulated among symptomatic patients. Our results add to those previous findings by incorporating analyses that demonstrate the importance of the adipocytokine signaling pathway of which these genes are members, and by demonstrating the cellular origin of the increased plaque levels of FABP4 and leptin.

Fatty-acid binding-protein 4 (FABP4 or adipocyte/macrophage fatty-acid binding-protein) was the gene most strongly upregulated in ruptured plaques in our microarray experiment. FABP4 is a member of the lipid chaperone family. It is expressed in adipocytes, macrophages and dendritic cells. In the macrophage, FABP4 attenuates cholesterol efflux via its inhibitory effect on the peroxisome proliferator-activated receptor gamma (PPARG) – liver X receptor-alpha (LXRa) – ATP binding cassette A1 (ABCA1) pathway [22]. It also regulates inflammatory responses via the inhibitor of kappa-B kinase (IKK)/JNK pathway [22]. FABP4 is the obligatory mediator coupling lipid-induced toxicity to endoplasmic reticulum (ER) stress; macrophages are particularly vulnerable to ER stress-associated apoptosis, especially in environments of high lipid exposure such as in the atherosclerotic plaque [23,24]. FABP4 plays a mechanistic role in ER stress through its regulation of fatty acid synthase (Fasn) and steaoryl CoA desaturase (SCD) via inhibition of the LXRa nuclear receptor [25]. Mice that are double knockout for Apolipoprotein E (APOE) and FABP4 are protected from atherosclerosis compared to the APOE knockout alone [26]; bone marrow transplantation studies have shown that this atheroprotective effect is mainly due to the absence of FABP4 in macrophages [26]. Macrophages from FABP4 knockout animals produce less pro-inflammatory cytokines [22]. In healthy volunteers, increased circulating FABP4 and Leptin have been recently shown to be independently associated with increased vascular inflammation measured using fluorodeoxyglucose positron emission tomography (FDG-PET) [27]. A genetic polymorphism in the FABP4 promoter region results in diminished FABP4 expression in adipose tissue, and has been associated with reduced risk of diabetes and cardiovascular disease in man [28]. Chemical inhibition of FABP4 using the small molecule BMS309403 in the APOE knockout mouse markedly reduced atheroma, and in particular macrophage foam cell formation [29]. Recent studies in humans found that both high local FABP4 expression in whole plaques and circulating plasma levels of FABP4, are associated with vulnerable plaque phenotypes, plaque-related symptoms, and future adverse clinical cardiovascular events [30,31]. Our cell-specific data complements these other observations, and highlights the potential of FABP4 as a biomarker or therapeutic target in plaque instability.

Leptin is the principal adipocytokine; it is produced by adipocytes and plays a role in satiety signaling. Recent studies have demonstrated that leptin has several other actions: it is produced during inflammation, and can modulate the innate and adaptive immune responses [32]. A variety of potential actions of leptin in the pathogenesis of atherosclerosis have been proposed - leptin promotes endothelial dysfunction, smooth muscle cell proliferation, platelet aggregation and thrombosis, the production of inflammatory cytokines and the calcification of vascular smooth muscle cells. The role of leptin in determining cardiovascular risk is complex. Hyperleptinaemia is an independent risk factor for coronary artery disease and acute myocardial infarction [32,33]. By contrast, however, leptin demonstrates acute vasodilatory effects via endothelial cell dependent and independent mechanisms, which are in contradiction to the pro-inflammatory, thrombogenic, and atherogenic effects seen in other studies [34]. These observations may be explained by the chronic effects of leptin on the endothelium, by the uncoupling of eNOS with excessive oxidative stress and depletion of NO leading to a disruption of vascular homeostasis and significant endothelial dysfunction [33–35]. An uncommon polymorphism in the leptin gene has been associated with carotid intima-medial thickness, a marker of atherosclerosis, in man [36].

Leptin was clearly expressed in our macrophage-rich areas, and our analyses of publicly available macrophage gene expression data confirmed that others had also observed leptin to be expressed in macrophages. Leptin down-regulates the expression of PPARG in macrophages in vitro, an effect it has in common with FABP4 [37], and it acts as a potent macrophage chemoattractant. Leptin has effects on macrophage lipid accumulation that are of potential importance in atherosclerosis: macrophages treated with leptin form cytoplasmic lipid bodies and synthesize the inflammatory eicosanoid leukotriene B4 [38]. Macrophages from mice deficient in leptin (ob/ob) have a reduced capacity to accumulate cholesterol compared with wild type cells [39]. Macrophages in human atherosclerotic plaques also express the leptin receptor [32]. Although a number of previous studies have investigated the consequences of the exogenous application of leptin to macrophages, and the local paracrine and ‘vasocrine’ effects of leptin from perivascular adipose tissue has been hypothesised and studied [32,33,35], none has explored the potential importance of autocrine/paracrine signaling by macrophage-derived leptin. Our results suggest such signaling may promote intracellular lipid accumulation and inflammatory mediator production that predispose to plaque instability. The differences we observed in the expression of downstream signaling molecules in the leptin pathway such as RXR-alpha, STAT3, IRS, and Akt, reinforce the suggestion that this pathway is activated when plaques become unstable.

Certain limitations of this study merit comment. The laser micro-dissection approach we describe is technically challenging, and was therefore performed on only a limited number of samples. This limits the precision with which we can quantify the expression differences we have observed. Although our comparisons with publicly available expression profiles from macrophages clearly indicate the largely macrophage origin of our RNA, we cannot exclude a degree of contamination from other cell types intimately associated with the macrophage in the atheromatous plaque. Nor can our approach take account of heterogeneity within the macrophage population in any plaque studied. Since plaques are known to progress by a repeated process of rupture (mainly asymptomatic) and healing, and some of the ruptured samples were collected up to three months after a clinical stroke or TIA, we also cannot entirely rule out an involvement of certain of the genes we have identified in the healing rather than the rupture process (although this seems unlikely to be the case for FABP4 and leptin). Future studies will be necessary to characterize the downstream consequences of the up-regulation of FABP4 and leptin expression in unstable plaque macrophages. Also, systematic investigation of differentially expressed genes in other KEGG pathways we identified would be of substantial interest.

In conclusion, we have demonstrated significant differences between gene expression patterns in macrophages from stable and ruptured plaques. Our results provide further support that therapeutic efforts to down-regulate the PPAR/Adipocytokine signaling pathway involving FABP4 and leptin in plaque macrophages may be a useful plaque stabilization strategy.

## Sources of funding

This study was funded by the Wellcome Trust and the British Heart Foundation. B.K. holds a British Heart Foundation personal chair.

## Disclosures

A.D.M. has received honoraria and sat on advisory committees for the following companies: Codman, Novo Nordisc and Stryker. A.D.M. is also a director of the Newcastle Neurosurgery Foundation.

## Figures and Tables

**Table 1:** Demographics of patients included in unstable and stable sample groups and the corresponding significance between groups (SD – standard deviation, IQR – inter-quartile range, ns – not significant, yrs – years).

	Unstable	Stable	*p*-value
No of samples (males)	12 (8)	13 (9)	ns
Mean age (SD)	65.9 yrs (8.3)	66.2 yrs (8.0)	ns
Median time from last event (IQR)	42 days (30–180)	n/a	–
Mean maximal luminal diameter stenosis on MR angiogram (SD)	82.3% (11.1)	85.2%(12.8)	ns
Smoking (present or previous)	67%	62%	ns
Hypercholesterolemia	83%	100%	ns
Hypertension	83%	69%	ns
Diabetes	25%	23%	ns
Ischemic heart disease	8%	54%	0.002
Medication: aspirin/clopidogrel	100%	92%	ns
Medication: statin	83%	100%	ns
Medication: ACEi/ARB	67%	69%	ns
Medication: metformin	17%	23%	ns

## Footnotes

Appendix A Supplementary data related to this article can be found, in the online version, at http://dx.doi.org/10.1016/j.atherosclerosis.2012.09.037.
