# Supplementary material for: Increased expression of fatty acid binding protein 4 and leptin in resident macrophages characterises atherosclerotic plaque rupture
Source: Atherosclerosis. 2013 Jan;226(1):74–81. doi: 10.1016/j.atherosclerosis.2012.09.037 (PMC3566542; doi:10.1016/j.atherosclerosis.2012.09.037)
Supplement: Supplementary file 1 [file mmc1.docx]

# Supplemental Materials

## 1. Scout immunostaining and LMD


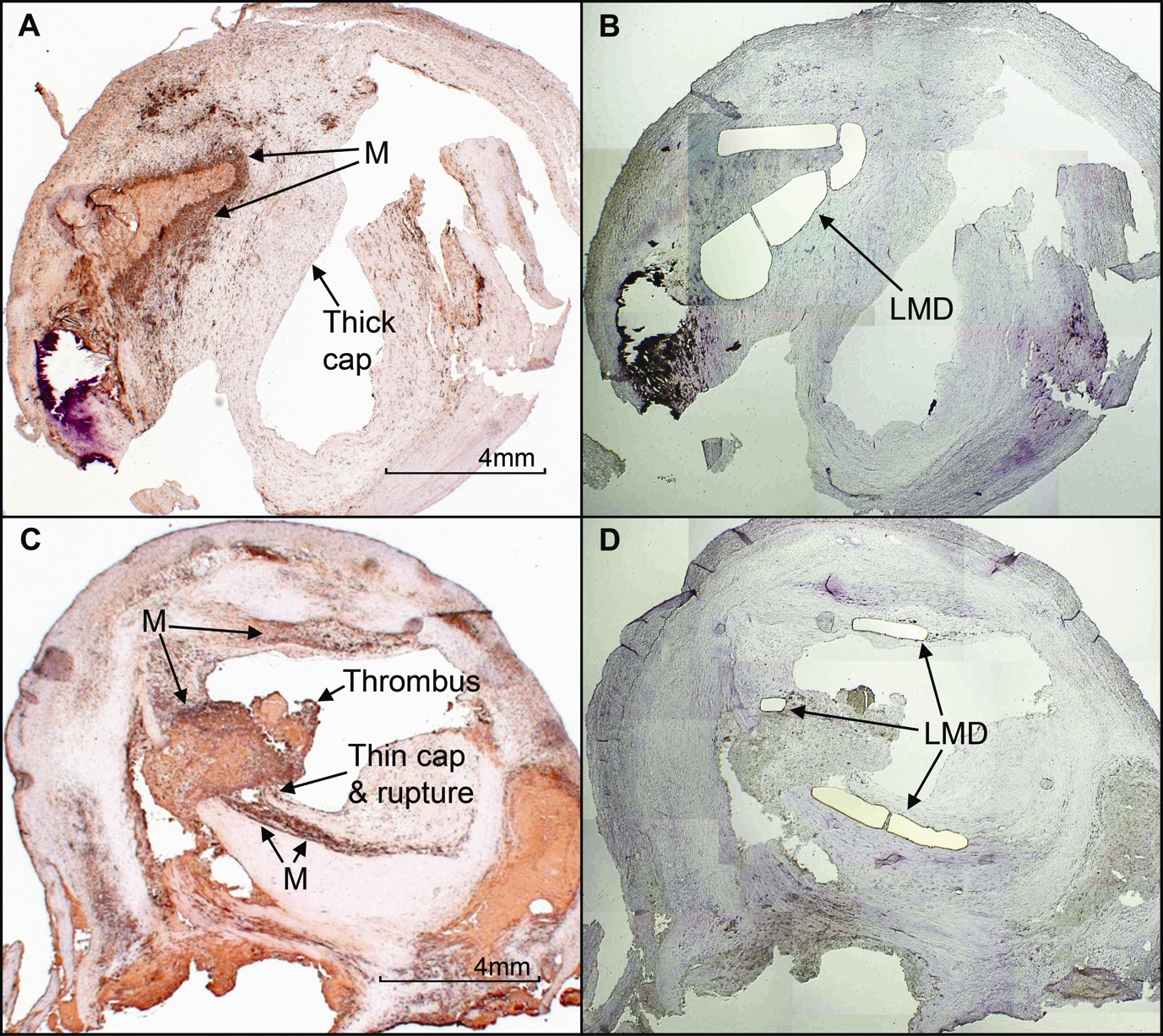


**Figure S1.** ‘Scout’ cryosections stained with CD68 to identify macrophage-rich regions (M) guiding laser micro-dissection on thermoplastic membranes of representative stable (panel A and B) and ruptured samples (panel C and D). The images in Panels B and D are a composite of stitched LMD images.

## 2. RNA quality


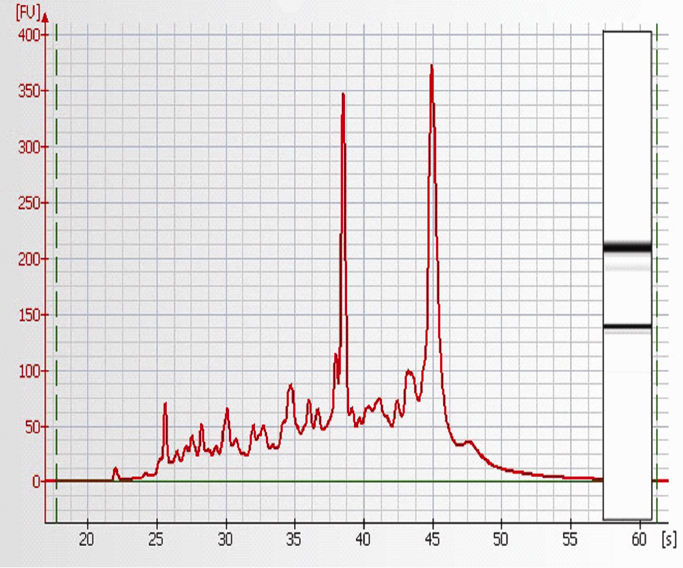


**Figure S2.** A representative Agilent Bioanalyser electropherogram of the extracted RNA shows high 28S and 18S peaks with no significant signal in the background.

## 3. Selection and validation of control genes

A compilation of 115 potential reference control genes was made from the Affymetrix 100 endogenous control genes mask (www.affymetrix.com/analysis/index.affx) and from commercially available control genes from Qiagen GeneGlobe and Taqman Primer-Probes which were represented on the HG-U133plus2 microarray. The top 7 genes that demonstrated the least variability in the GCRMA normalised microarray data were selected for further testing by qRTPCR (Table S1).

In order to identify the most stable reference control genes in our study population we used the method described by Vandesompele et al using the geNorm analysis software^1,2^. In summary, the expression stability was evaluated and a gene-stability measure (M) based on the average pairwise variation between a particular gene and all the other genes studied was generated. High expression stability is indicated by a low M value as an estimate of combined variation of the individual gene. The M values calculated by geNorm for the seven candidate reference controls are shown in Table S2. PPIA and SDHA were accordingly selected as reference genes.

| **Gene Symbol** | **Gene Name** | **Entrez Gene ID** | **Taqman Probe ID** |
| --- | --- | --- | --- |
| PPIA | Peptydylprolyl Isomerase A (Cyclophillin A) | 5478 | HS99999904_m1 |
| SDHA | Succinate Dehydrogenase Complex Subunit A | 6389 | HS00188166_m1 |
| GAPDH | Glyceraldehyde-3-phosphate dehydrogenase | 2597 | HS00266705_g1 |
| MYST2 | MYST histone acetyltransferase 2 | 11143 | HS00272972_m1 |
| GUSB | Glucuronidase, Beta | 2990 | HS99999908_m1 |
| TBP | TATA box binding protein | 6908 | HS00427620_m1 |
| TUBB | Tubulin, Beta polypeptide | 203068 | HS00742828_s1 |

**Table S1.** The shortlist of 7 candidate reference genes for further validation by qRT-PCR.

| **Ranking** | **Gene** | **M value** |
| --- | --- | --- |
| 1 | PPIA | 0.335 |
| 2 | SDHA | 0.360 |
| 3 | TUBB | 0.389 |
| 4 | TBP | 0.410 |
| 5 | MYST2 | 0.500 |
| 6 | GAPDH | 0.534 |
| 7 | GUSB | 0.603 |
| Best combination of 2 genes | PPIA and SDHA | 0.142 |

**Table S2.** Expression stability of the candidate reference genes calculated by the M-statistic, and ranked from the most stable at the top. Low M values indicate high expression stability.

## 4. Real-time quantitative PCR primers

| **Gene Symbol** | **Gene Name** | **EntrezGene ID** | **Taqman Probe ID** |
| --- | --- | --- | --- |
| PPIA | Peptydylprolyl Isomerase A (Cyclophillin A) | 5478 | HS99999904_m1 |
| SDHA | Succinate Dehydrogenase Complex Subunit A | 6389 | HS00188166_m1 |
| FABP4 | Fatty Acid Binding Protein 4 | 2167 | HS00609791_m1 |
| PPBP | Pro-Platelet Binding Protein 4 | 5473 | HS00234077_m1 |
| PACAP | Pro-Apoptotic Caspase Adaptor Protein | 51237 | HS00414907_m1 |
| FCGR3AB | Fc Fragment of IgG, low affinity IIIA & IIIB Receptor (CD16a&b) | 2214/2215 | HS00275547_m1 |
| LEP | Leptin | 3952 | HS00174877_m1 |
| CD38 | CD38 antigen (p45) | 952 | HS00277045_m1 |
| PDGFD | Platelet Derived Growth Factor D | 80310 | HS00228671_m1 |
| ECM2 | Extracellular Matrix Protein 2 | 1842 | HS00154821_m1 |
| RGS5 | Regulation of G-Protein Signalling 5 | 8490 | HS00186212_m1 |
| OGN | Osteoglycin | 4969 | HS00247901_m1 |
| SFRP2 | Secreted Frizzled-Related Protein 2 | 6423 | HS00293258_m1 |
| ASPN | Asporin | 54829 | HS00214395_m1 |

**Table S3.** Real-time quantitative PCR primer-probe details.

5. Principal Components Analysis of Genes of Macrophage Activation

The principal components analysis comparing only the expression of genes previously shown by others to be involved in macrophage activation, obtained from the paper by Cho et al (3815 genes)^3^, show that both the stable and ruptured LMD samples overlap and cluster with the activated macrophages studied by others^3, 5-7^ (Figure S3, orange clusters). Moreover, they are separate from the expression profiles of unactivated differentiated macrophages^3^ (blue clusters), differentiated monocytes and peripheral blood monocytes (PBMCs) from various previous studies^3, 4, 6^ (green clusters). They are also distant from the other inflammatory cell types (T-lymphocyte and B-lymphocyte cells, natural killer (NK) cells)^4^ and whole peripheral blood cells^4^ (green clusters). This demonstrates that the gene expression profiles in our laser micro-dissected samples closely resemble those described in the literature to date for activated macrophages.


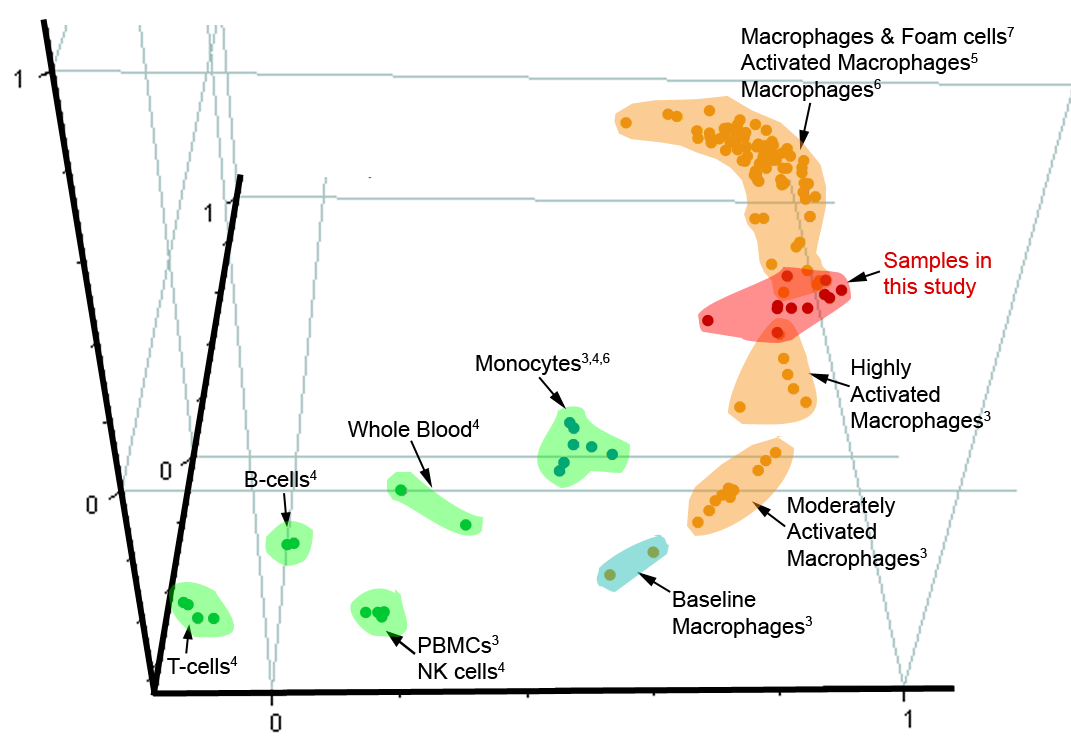


**Figure S3.** Principal Components Analysis of laser micro-dissected samples in this study with samples of blood cell origin and activated/unactivated macrophages using the expression profiles of a subset of genes involved in macrophage activation.

## 6. KEGG Pathways

| KEGG Parent Node | KEGG Pathway | Observed no. of genes in genelist | Total no. of genes in pathway | P-value | Group with majority genes up-regulated (no. of genes) |
| --- | --- | --- | --- | --- | --- |
| Cell Communication | [Focal adhesion](http://www.genome.ad.jp/dbget-bin/show_pathway?hsa04510+10000+1278+1290+2335+23533+2534+29780+3685+3688+3912+3915+394+399694+4233+4638+5156+5728+5747+64098+7058+7414+80310+8515+858+9475) | 25 | 203 | 1.30x10^-10^ | Stable(22) |
| Cell Communication | [Adherens junction](http://www.genome.ad.jp/dbget-bin/show_pathway?hsa04520+10458+10580+117178+2534+25945+4008+4233+56288+5787+5792+6591+6934+7082+7414+7454+91) | 16 | 78 | 1.41x10^-10^ | Stable(13) |
| Cell Motility | Regulation of actin cytoskeleton | 24 | 217 | 2.62x10^-9^ | Stable(16) |
| Signal Transduction | PPAR/Adipocytokine signaling Pathway | 15 | 121 | 5.45x10^-7^ | Ruptured(11) |
| Signaling Molecules and Interaction | [ECM-receptor interaction](http://www.genome.ad.jp/dbget-bin/show_pathway?hsa04512+1278+1290+2335+3685+3688+3912+3915+7058+84624+8515+961) | 11 | 85 | 1.12x10^-5^ | Stable(11) |
| Cell Communication | [Tight junction](http://www.genome.ad.jp/dbget-bin/show_pathway?hsa04530+10000+154810+2036+22800+4628+50848+51776+5524+56288+5728+7082+8573+9414) | 13 | 135 | 4.36x10^-5^ | Stable(10) |
| Signaling Molecules and Interaction | [Cytokine-cytokine receptor interaction](http://www.genome.ad.jp/dbget-bin/show_pathway?hsa04060+1441+1524+2920+2921+355+3572+3587+3588+3952+3977+4233+5156+5473+657+659+7852+91+939+958) | 19 | 263 | 4.89x10^-5^ | Balanced  (11 ruptured) |
| Signal Transduction | [Insulin signaling pathway](http://www.genome.ad.jp/dbget-bin/show_pathway?hsa04910+10000+10580+23433+23533+3667+369+399694+5562+5792+6194+6720+8569) | 12 | 138 | 2.11x10^-4^ | Balanced  (7 stable) |
| Signal Transduction | [TGF-beta signaling pathway](http://www.genome.ad.jp/dbget-bin/show_pathway?hsa04350+10468+4052+57154+5934+657+659+7058+91+9475) | 9 | 87 | 3.60x10^-4^ | Stable(8) |
| Signaling Molecules and Interaction | [Cell adhesion molecules](http://www.genome.ad.jp/dbget-bin/show_pathway?hsa04514+1000+25945+3685+3688+4756+50848+5792+7412+920+941+958) | 11 | 133 | 5.61x10^-4^ | Stable(7) |

**Table S4.** The list of statistically significant KEGG Pathways identified using WebGestalt.

## 7. PPAR/Adipocytokine Signaling Pathway

The PPAR/Adipocytokine Signaling Pathway, which incorporates FABP4 and Leptin, was the most strongly up-regulated KEGG pathway in unstable samples (p=5.5x10^-7^). Fifteen genes were identified to be significantly differentially expressed between stable and unstable plaques, of which eleven genes were up-regulated in the unstable plaque. The details of these genes are shown in Table S5. The false discovery rate and fold change were from the microarray data of 11 samples.

| **Gene Name** | **Gene Symbol** | **Entrez-GeneID** | **Regulation** | **FDR** | **Fold Change** |
| --- | --- | --- | --- | --- | --- |
| Fatty acid binding protein 4, adipocyte/macrophage | FABP4 | 2167 | Up-regulated | 0.072 | 28.1 |
| Leptin | LEP | 3952 | Up-regulated | 0.071 | 6.61 |
| Acyl-CoA synthetase long-chain family member 1 | ACSL1 | 2180 | Up-regulated | 0.067 | 3.02 |
| Cytochrome P450, family 27, subfamily A, polypeptide 1 | CYP27A1 | 1593 | Up-regulated | 0.094 | 2.96 |
| Phospholipid transfer protein | PLTP | 5360 | Up-regulated | 0.093 | 2.93 |
| Glycerol kinase | GK | 2710 | Up-regulated | 0.084 | 2.16 |
| Retinoid X receptor, alpha | RXRA | 6256 | Up-regulated | 0.060 | 2.14 |
| Signal transducer and activator of transcription 3 (acute-phase response factor) | STAT3 | 6774 | Up-regulated | 0.077 | 2.10 |
| Acyl-CoA synthetase long-chain family member 5 | ACSL5 | 51703 | Up-regulated | 0.069 | 1.80 |
| TNFRSF1A-associated via death domain | TRADD | 8717 | Up-regulated | 0.089 | 1.55 |
| Acetyl-Coenzyme A acyltransferase 1 | ACAA1 | 30 | Up-regulated | 0.062 | 1.44 |
| Protein kinase, AMP-activated, alpha 1 catalytic subunit | PRKAA1 | 5562 | Down-regulated | 0.056 | 1/1.63 |
| V-AKT murine thymoma viral oncogene homolog 3 (protein kinase B, gamma) | AKT3 | 10000 | Down-regulated | 0.067 | 1/2.70 |
| Insulin receptor substrate 1 | IRS1 | 3667 | Down-regulated | 0.072 | 1/5.49 |
| Sorbin and SH3 domain containing 1 | SORBS1 | 10580 | Down-regulated | 0.075 | 1/10.9 |

**Table S5.** Differentially regulated genes in the PPAR/Adipocytokine Signalling Pathway (False Discovery Rate; FDR<10%).

## References

**1.** Hellemans J, Mortier G, De Paepe A, Speleman F, Vandesompele J. qBase relative quantification framework and software for management and automated analysis of real-time quantitative PCR data. *Genome Biol.* 2007;8(2):R19.

**2.** Vandesompele J, De Preter K, Pattyn F, Poppe B, Van Roy N, De Paepe A, Speleman F. Accurate normalization of real-time quantitative RT-PCR data by geometric averaging of multiple internal control genes. *Genome Biol.* 2002;3(7):RESEARCH0034.

**3.** Cho HJ, Shashkin P, Gleissner CA, Dunson D, Jain N, Lee JK, Miller Y, Ley K. Induction of dendritic cell-like phenotype in macrophages during foam cell formation. *Physiological genomics.* 2007;29(2):149-160.

**4.** Su AI, Wiltshire T, Batalov S, Lapp H, Ching KA, Block D, Zhang J, Soden R, Hayakawa M, Kreiman G, Cooke MP, Walker JR, Hogenesch JB. A gene atlas of the mouse and human protein-encoding transcriptomes. *Proceedings of the National Academy of Sciences of the United States of America.* 2004;101(16):6062-6067.

**5.** Jura J, Wegrzyn P, Korostynski M, Guzik K, Oczko-Wojciechowska M, Jarzab M, Kowalska M, Piechota M, Przewlocki R, Koj A. Identification of interleukin-1 and interleukin-6-responsive genes in human monocyte-derived macrophages using microarrays. *Biochimica et biophysica acta.* 2008;1779(6-7):383-389.

**6.** Liu H, Shi B, Huang CC, Eksarko P, Pope RM. Transcriptional diversity during monocyte to macrophage differentiation. *Immunology letters.* 2008;117(1):70-80.

**7.** Hagg DA, Jernas M, Wiklund O, Thelle DS, Fagerberg B, Eriksson P, Hamsten A, Olsson B, Carlsson B, Carlsson LM, Svensson PA. Expression profiling of macrophages from subjects with atherosclerosis to identify novel susceptibility genes. *International journal of molecular medicine.* 2008;21(6):697-704.
